# Macroevolutionary thermodynamics: Temperature and the tempo of evolution in the tropics

**DOI:** 10.1371/journal.pbio.3001368

**Published:** 2021-08-25

**Authors:** Daniel L. Rabosky

**Affiliations:** Museum of Zoology & Department of Ecology and Evolutionary Biology, University of Michigan, Ann Arbor, Michigan, United States of America

## Abstract

An influential hypothesis proposes that the tempo of evolution is faster in the tropics. Emerging evidence, including a study in this issue of *PLOS Biology*, challenges this view, raising new questions about the causes of Earth’s iconic latitudinal diversity gradient (LDG).

Biologists have long puzzled over the spectacular diversity of animals and plants from Earth’s tropical regions. It is true that some tropical environments are not especially rich in species, and some groups of organisms show contrarian patterns with diversity peaks that occur far outside of the warm, humid tropics. Nonetheless, the big picture is clear: A vastly disproportionate fraction of Earth’s terrestrial biodiversity is concentrated in tropical rainforests, and warm water reef environments similarly account for a large fraction of marine diversity. The extremes of tropical diversity transcend the ability of most humans to process it: Some Amazonian rainforests, for example, contain more species of trees in just a few hectares than are found in the entirety of Europe or North America [1]. In general, the most diverse tropical rainforests support order-of-magnitude increases in species richness relative to otherwise comparable temperate zone communities across a wide range of organisms. Despite decades of study, however, the causes of this latitudinal diversity gradient (LDG) remain elusive.

One of the most prominent hypotheses for the LDG is, loosely speaking, the idea that biological processes speed up in the tropics, potentially due to the kinetic effects of temperature on the rates of organismal processes. It seems obvious that the pulse of life should be faster under a torrid tropical sun, and—to naturalists who’ve spent time in lowland rainforests in particular—such a view accords well with perceptions of the humid tropics as a raging, steamy mess of species interactions that collectively generate the tangled web that is tropical diversity. It is generally accepted that temperature can affect metabolic rate and many other biological processes, including those involving ecological interactions between species (e.g., competition, predation, and herbivory). The specific mechanisms connecting thermal energy to biodiversity remain unclear. For example, they might involve the influence of temperature on rates of molecular evolution, which might then influence rates of speciation [2]. Or, species in warmer environments might live closer to their optimal body temperatures, thus enabling them to allocate more resources to performance-associated functions and leading to a systematic upgrading in the intensity of antagonistic or coevolutionary interactions between species [3]. Regardless of the specific mechanism, the general idea is captured by Brown [4], who notes that “‘Diversity begets diversity’ in the tropics, because ‘the Red Queen runs faster when she is hot.’”

Writing in *PLOS Biology*, Drury and colleagues [5] demonstrate that a central prediction of these “faster tropics” hypotheses fails to hold up. They predicted that, if certain types of ecological interactions between species are stronger in the tropics, then we should see a signal of those interactions in long-term patterns of trait evolution. In particular, the increased pressure to respond to species interactions in the tropics should result in faster overall rates of morphological evolution for tropical species. To test this hypothesis, the authors studied the rate of morphological evolution in birds, analyzing a large dataset on bill shape and body proportions from other recent studies [6] with a battery of sophisticated statistical models. These models allowed the rate of morphological change to differ systematically with latitude. Intriguingly, the models that best fit the data in some cases were those that allowed for strong interactions between species in driving patterns of divergence among closely related species that occupied that same biogeographic region (e.g., the neotropics). Thus, there is a partial signal of species interactions on the morphologies of species we see living together today, including those from both tropical and temperate regions. As suggested by the authors, these patterns might reflect a form of ecological character displacement, whereby morphologically similar species evolve differences that minimize their ecological overlap. But, surprisingly, the intensity of these effects shows no consistent relationship with latitude. The take-home message is that—at least for birds and the traits considered—species are not evolving more rapidly in the tropics.

Drury and colleagues note that their results contradict recent articles that have documented differences in phenotypic evolutionary rates across latitude, although the studies referenced generally looked at different types of traits (e.g., birdsong). They suggest several potential reasons for the discrepancies between their results and those prior studies. But, critically, these earlier studies generally did not report faster evolution in the tropics, but *faster evolution in the temperate zone*. Hence, the results of Drury and colleagues and the earlier studies all converge to a similar and more general finding, which is that the warm tropics really aren’t so hot for macroevolution, at least as far as phenotypic evolutionary rates are concerned. By rejecting the simple explanations (faster evolution), new questions emerge about how and why tropical bird communities show such dramatic phenotypic and ecological diversity.

Morphological evolution is not the only process that fails to show the expected pattern of “heating up” in the tropics. A number of recent studies have found that rates of species formation are either unrelated to latitude or slower in the tropics [7–9]. These results argue strongly against temperature kinetic models of biodiversity, whereby faster speciation emerges from the effects of warmer temperatures in the tropics on mutation and metabolic rates [10]. Many of the same causal pathways that predict increased rates of speciation as a function of temperature would also apply to rates of morphological evolution: Increased mutation rates in the tropics, for example, should accelerate the tempo of phenotypic evolution due to increased mutational variance in traits. But, regardless of whether we consider phenotypic evolution (as in Drury and colleagues) or lineage diversification, there is simply no evidence for faster evolutionary rates in the tropics.

The results from Drury and colleagues [5] and other studies do not reject all possible causal pathways by which temperature or species interactions might facilitate high tropical diversity. Many phylogeny-based studies of species diversification and phenotypic evolution frame their interpretations through the lens of interspecific competition, ecological opportunity, and character displacement. Yet, numerous other types of interactions are relevant to global biodiversity patterns, and some of these interactions have scarcely been explored from a macroevolutionary perspective. Many such interactions have the potential to influence species richness and ecological diversity, perhaps through mechanisms that involve an indirect effect of temperature on equilibrium diversity levels. With more data on how host–pathogen, predator–prey, and other biotic interactions vary latitudinally, perhaps we will emerge with a greater understanding of the diverse mechanisms that contribute to the spectacular enrichment of tropical diversity.

## References

[1] Bass MS, Finer M, Jenkins CN, Kreft H, Cisneros-Heredia DF, McCracken SF, et al. Global conservation significance of Ecuador’s Yasuní National Park. PLoS ONE. 2010;5:e8767. doi: 10.1371/journal.pone.0008767 20098736 PMC2808245

[2] Rohde K. Latitudinal diversity gradient in species diversity: the search for the primary cause. Oikos. 1992;65:514–27.

[3] Schemske DW. Biotic interactions and speciation in the tropics. In: Butlin RK, Bridle JR, Schluter D, editors. Speciation and patterns of diversity. Cambridge University Press; 2009. p. 219–39.

[4] Brown JH. Why are there so many species in the tropics? J Biogeogr. 2014;41:8–22. doi: 10.1111/jbi.12228 25684838 PMC4320694

[5] Drury J, Clavel J, Tobias JA, Rolland J, Sheard C, Morlon H. Tempo and mode of morphological evolution are decoupled from latitude in birds. PLoS Biol. 2021. doi: 10.1371/journal.pbio.3001270 PMC8384433 34428214

[6] Pigot AL, Sheard C, Miller ET, Bregman TP, Freeman BG, Roll U, et al. Macroevolutionary convergence connects morphological form to ecological function in birds. Nat Ecol Evol. 2020;4:230–9. doi: 10.1038/s41559-019-1070-4 31932703

[7] Igea J, Tanentzap AJ. Angiosperm speciation cools down in the tropics. Ecol Lett. 2020;23:692–700. doi: 10.1111/ele.13476 32043734 PMC7078993

[8] Rabosky DL, Chang J, Title PO, Cowman PF, Sallan L, et al. An inverse latitudinal gradient in speciation rate for marine fishes. Nature. 2018;559:392–5. doi: 10.1038/s41586-018-0273-1 29973726

[9] Harvey MG, Bravo GA, Claramunt S, Cuervo AM, Derryberry GE, Battilana J, et al. The evolution of a tropical biodiversity hotspot. Science. 2020;370:1343–8. doi: 10.1126/science.aaz6970 33303617

[10] Worm B, Tittensor DP. A theory of global biodiversity. Princeton University Press; 2018.

